# Afterload excess and myocardial performance

**DOI:** 10.1186/1532-429X-15-S1-P220

**Published:** 2013-01-30

**Authors:** Meghana Jayam, Danielle Janosevic, Madhavi Kadiyala, Jie J Cao, Simcha Pollack, Nathaniel Reichek

**Affiliations:** 1 Research, St. Francis Hospital, Roslyn, NY, USA

## Background

Systolic circumferential and longitudinal strain(CSt%, LSt%) are widely used to assess myocardial performance, but their afterload dependence has not been well characterized. Using geometric estimates of left ventricular (LV) circumferential and meridional wall stress(CWS, MWS) as indices of afterload at the myocardial level, we compared LV CSt and LSt to estimates of CWS and MWS in normals (NL, n= 39, 46% female, age 54.6+/-14.6 yrs) and patients with dilated cardiomyopathy (DCM, n= 35, 23% female, age 50.8+/-15.0 yrs, EF 27.2+/-10.8%).

## Methods

Breath-hold retrospectively gated SSFP cine images in short and long axis planes and cuff systolic blood pressure(P) during imaging were obtained and mid LV and "global" (average of basal, midLV and apical) CSt% and LSt% determined using feature tracking (2D CPA MR, TomTec Imaging Systems, Munich). Mid-LV CWS and MWS were estimated using Mirsky's published formulae (CWS= P(B/h)[1 - (B2/2A2) - (h/2b); where h=end-systolic(ES) LV wall thickness, A= ES midwall semimajor axis ([L + h]/2), B= ES midwall semiminor axis ([D + h]/2)) and MWS= PRi/(2h(1+h/2Ri) where Ri= short axis LV radius).

## Results

Mean mid-LV CSt% was -22.2+/-4.8% in NL but markedly reduced in DCM (-9.3+/-5.0%, p< 0.0001) while mean mid-LV LSt% was -14.7/-+8.9 in NL and markedly reduced in DCM(-8.6+/-4.6, p< 0.0006). Reductions in CSt% and LSt% in DCM were associated with markedly elevated CWS (307.6+/-9.2 vs 176.2+/-42.1x103dyn/cm2 in NL, p<0.0001) and MWS(195.6+/-69.6 vs. 92.0+/-25.7 x103dyn/cm2, p<0.001). Similar results were obtained for "global" CSt% and LSt%, the means of CSt% and LSt% at basal, mid-LV and apical slice locations(Table 1) and for MWS versus LV long axis shortening. The relationship between CWS and mid-LV CSt% was CSt% = 0.055CWS-29.3, r= -0.64, p< 0.001. Therefore, if NL LV myocardium were exposed to the same afterload(CWS) as in DCM, mean CST% might fall from -22.3% to -12.1%. Similar effects would be seen on LSt% under the markedly increased MWS found in DCM. Thus, afterload excess due to adverse remodeling and noncompensatory(inadequate) hypertrophy could account for as much as 79% of the reduction in strain in DCM, and altered myocyte "contractile state" and altered myocardial composition for as little as 21% of the deficit.

**Table 1:** Global Circumferential and Longitudinal Strains and Wall Stress

	Normal n=39	DCM n=35	t-test
Variable	mean(s.d.)	mean(s.d.)	p-value

Global Circ Strain(%)	-23.8(4.3)	-10.7(5.3)	<0.0001
Circ Wall Stress x10^3^dynes/cm2	176.2(42.1)	307.6(89.2)	<0.0001
Global Long. Strain(%)	-16.2(4.5)	-8.6(4)	<0.0001
Meridional Stressx10^3^dynes/cm2	92.0(25.7)	195.6(69.6)	<0.0001

## Conclusions

We conclude that afterload excess is a major contributor to impaired systolic function in DCM and normalization of contractile state and myocardial composition alone may not restore normal function. Consideration of effects of afterload excess, inadequate hypertrophy and adverse remodeling on myocardial strain is essential in the evaluation of pathophysiology.

## Funding

This study was supported by the St. Francis Research Foundation

